# Supplementary material for: Whole Genome Sequencing of Field Isolates Reveals Extensive Genetic Diversity in Plasmodium vivax from Colombia
Source: PLoS Negl Trop Dis. 2015 Dec 28;9(12):e0004252. doi: 10.1371/journal.pntd.0004252 (PMC4692395; doi:10.1371/journal.pntd.0004252)
Supplement: S1 Text — (DOCX) [file pntd.0004252.s001.docx]

**Oligonucleotide primers for polymorphic microsatellite DNA markers**

Primers were tested on a panel of 6 positive samples for *P. vivax* from two Colombian localities: 3 from Tierralta (Department of Cordoba in the northern area of Colombia), and 3 from Tumaco (Department of Nariño in the southeast area of the Pacific Coast). *Plasmosdium vivax* strain Salvador I was used as a positive control. Polymerase chain reactions (PCRs) was carried out in a 15 µL volume reaction using 2 µL of total genomic DNA, 0.6 µL of each primer (2.5 µM), 7.5 µL of PCR Master Mix (2X) from Promega (50 units/ml of Taq DNA polymerase in a reaction buffer (pH 8.5), 3.5 mM MgCl2, and 400µM of each deoxynucleoside triphosphate (dATP, dGTP, dCTP, dTTP) and 4.3 µL of water. The PCR program conditions were: a partial denaturation at 94ºC for 4 min and 40 cycles with 30 sec at 94ºC, 40 sec at 54ºC (for the annealing temperature see Supplementary Table XB) and 30 sec extension at 72ºC. A final extension of 5 min at 72ºC was added in the last cycle. Fluorescently labelled PCR products were separated on an Applied Biosystems 3730 capillary sequencer and the microsatellite peak heights were scored using GeneMarker V2.6.3. (SoftGenetics LLC). All microsatellite markers amplified and out of 18, 16 were polymorphic in these small data set (6 samples plus Strain Salvador I); however all loci were polymorphic in the Colombian samples genomes obtained in this study The length of allele range in base pairs (BP) is given in the last column in S1 Table. As an example, the electropherograms for one sample for each locus is shown in S1 Figure. For the polymorphic microsatellites, between 2 and 4 alleles were found per locus.
