# Supplementary material for: Whole Genome Sequencing of Field Isolates Reveals Extensive Genetic Diversity in Plasmodium vivax from Colombia
Source: PLoS Negl Trop Dis. 2015 Dec 28;9(12):e0004252. doi: 10.1371/journal.pntd.0004252 (PMC4692395; doi:10.1371/journal.pntd.0004252)
Supplement: S1 Table — (DOCX) [file pntd.0004252.s002.docx]

| **Sample** | **Age (years)** | **Parasitemia (parasites/µL)** | **Days of patency** | **Medical History** |
| --- | --- | --- | --- | --- |
| 446 | 22 | 6200 | 4 | Chloroquine/primaquine 3 months before the current episode |
| 494 | 19 | 6486 | 1 | Chloroquine/ primaquine 12 months before the current episode |
| 495 | 13 | 9000 | 1 | None |
| 496 | 12 | 14980 | 1 | None |
| 498 | 10 | 5600 | 1 | None |
| 499 | 26 | 12320 | 1 | Hospitalized during the current episode, chloroquine/ primaquine 12 months before the current episode |
| 500 | 18 | 8400 | 1 | None |
| 503 | 16 | 9142 | 1 | None |
