# Supplementary material for: Whole Genome Sequencing of Field Isolates Reveals Extensive Genetic Diversity in Plasmodium vivax from Colombia
Source: PLoS Negl Trop Dis. 2015 Dec 28;9(12):e0004252. doi: 10.1371/journal.pntd.0004252 (PMC4692395; doi:10.1371/journal.pntd.0004252)
Supplement: S2 Table — Size ranges of PCR products (in base pairs) are given for six of the Colombian P. vivax isolates. Sal-I was used as a positive control. Fluorescent dyes (Hex and 6-FAM) were used to label forward primers only. ML: Motif length and No.A: allele numbers. (DOCX) [file pntd.0004252.s003.docx]

**S1 Table**

| **Chr** | **Name** | **ML** | **motif** | **Length^a^** | **Name** | **Primer Forward (5’-3’)** | **Name** | **Primer Reverse (5’-3’)** | **Annealing Temp.**  **(ºC)** | **Allele**  **Range (bp)** | **No. Alleles** |
| --- | --- | --- | --- | --- | --- | --- | --- | --- | --- | --- | --- |
| 2 | CLAIM1 | 4 | ATGC | 120 | 2_96021F | **HEX**-TGC AAA TAG GTA CAT AGG TAT G | 2_96021R | AGT TGG AAA GGA GTT GCA C | 56.4/55 | 116-128 | 4 |
| 2 | CLAIM2 | 2 | AC | 168 | 2_261105F | **HEX**-CCC TCA TCT TGG TAA ATC TG | 2_261105R | CAT TTT AAA CAG CCT GCA CC | 56.4/56.4 | 164-185 | 4 |
| **5*** | CLAIM3 | 6 | AACAGC | 187 | 5_280204F | **FAM** -AAC ACC GTT TTG AGG AAC G | 5_280204R | GCT CTG CTA AGC ACA TTA AAC | 55/57.5 | 169-187 | 3 |
| **5*** | CLAIM4 | 2 | AT | 192 | 5_1139547F | **FAM**-TTT GAG GAC AGA ATG CGT AC | 5_1139547R | ATT ATG TAT TTC CCT CTT TCT C | 56.4/54.7 | 192-200 | 4 |
| **5*** | CLAIM5 | 4 | AAAT | 193 | 5_1195225F | **FAM**-CCA TCT CAT TTG GCT AAT CG | 5_1195225R | AAG ACA GAT GTG CAC TTT GG | 56.4/56.4 | 185-205 | 4 |
| 5 | CLAIM6 | 2 | AT | 183 | 5_1248584F | **FAM**-CAT TTC GAC AAG CAA GCA TG | 5_1248584R | TGT GGT GAA CTT ATG TGT GG | 56.4/56.4 | 183-185 | 2 |
| 6 | CLAIM7 | 2 | AT | 177 | 6_436676F | **FAM**-AGT GCT GTA TAG ATA CAA AAG C | 6_436676R | TTT AAC TAG TTA ATA ACT GGT TGC | 56.4/56.6 | 177 | 1 |
| **7*** | CLAIM8 | 2 | AT | 131 | 7_761767F | **HEX**-ACA CAT ACA TAC ATA TAT ATA ATA C | 7_761767R | TAC AAG GTG AGC AGT TTG AG | 54.3/56.4 | 131-135 | 2 |
| 7 | CLAIM9 | 2 | AG | 107 | 7_1343057F | **HEX**-GTA TGG TGT AAG TGG TGT TC | 7_1343057R | ATT GCA CTC TCG TGG ATA AG | 56.4/56.4 | 107 | 1 |
| **8*** | CLAIM10 | 2 | AT | 340 | 8_439431F | **HEX**-AGC TTC ACT ATA TTG CCT CC | 8_439431R | CTA CGT CTG ACA TTT CGA TC | 56.4/56.4 | 336-340 | 4 |
| 9 | CLAIM11 | 4 | AAAT | 263 | 9_1048573F | **FAM**-TAA ATC GAG TTA TAC AGC AGC | 9_1048573R | GAA GGA ACA AAT ACT GCT GG | 55.4/56.4 | 259-267 | 4 |
| **9*** | CLAIM12 | 2 | AT | 278 | 9_1454922F | **FAM**-TAA GGA AAT GCA CAG GTA GG | 9_1454922R | GCT GAC CTT CTA TTT GAT GG | 56.4/56.4 | 276-278 | 2 |
| **10*** | CLAIM13 | 2 | AT | 100 | 10_1348254F | **HEX**-TTC GTT AGA AGA CAA AAC TGG | 10_1348254R | AAA GTA CGT TGG TTT TAC AG | 55.4/52.3 | 100-102 | 2 |
| 10 | CLAIM14 | 2 | AT | 118 | 10_1368121F | **HEX**-AAG ATG CTT TAA AAA TAG GTA AAT C | 10_1368121R | CGC GGA ATA TGT TCA ATG TG | 55.9/56.4 | 116-118 | 3 |
| 12 | CLAIM15 | 2 | AC | 148 | 12_2076009F | **HEX**-TGC ATA TGC GTG TGC GTT C | 12_2076009R | CCA GGA TTT CAT TAA CAT CGG | 57.5/57.5 | 144-152 | 3 |
| **12*** | CLAIM16 | 2 | AT | 161 | 12_2101147F | **HEX**-CGG GGA ATG TGT TAT GTG C | 12_2101147R | TAA GTC TGT GCC TCA TAG G | 57.5/55 | 161-163 | 2 |
| **13*** | CLAIM17 | 2 | AT | 201 | 13_950389F | **FAM**-TTC TCT TCA GAC ATC ATT TAG G | 13_950389R | CCT TAA CGG TGA AGT TGT C | 56.4/55 | 199-201 | 2 |
| 14 | CLAIM18 | 2 | AT | 211 | 14_1469652F | **FAM**-ATC TGT TCG ATA AGC TGA GG | 14_1469652R | ACA TTC GAT TGA CTC CTA CG | 56.4/56.4 | 211-221 | 3 |
| ***** Conserved regions between *P. vivax* and *P. cynomolgi*  **^a^** Total PCR product length for *P. vivax* strain Salvador I. | | | | | |  |  |  |  |  |  |
