# Supplementary figures and images for: Whole Genome Sequencing of Field Isolates Reveals Extensive Genetic Diversity in Plasmodium vivax from Colombia
Source: PLoS Negl Trop Dis. 2015 Dec 28;9(12):e0004252. doi: 10.1371/journal.pntd.0004252 (PMC4692395; doi:10.1371/journal.pntd.0004252)

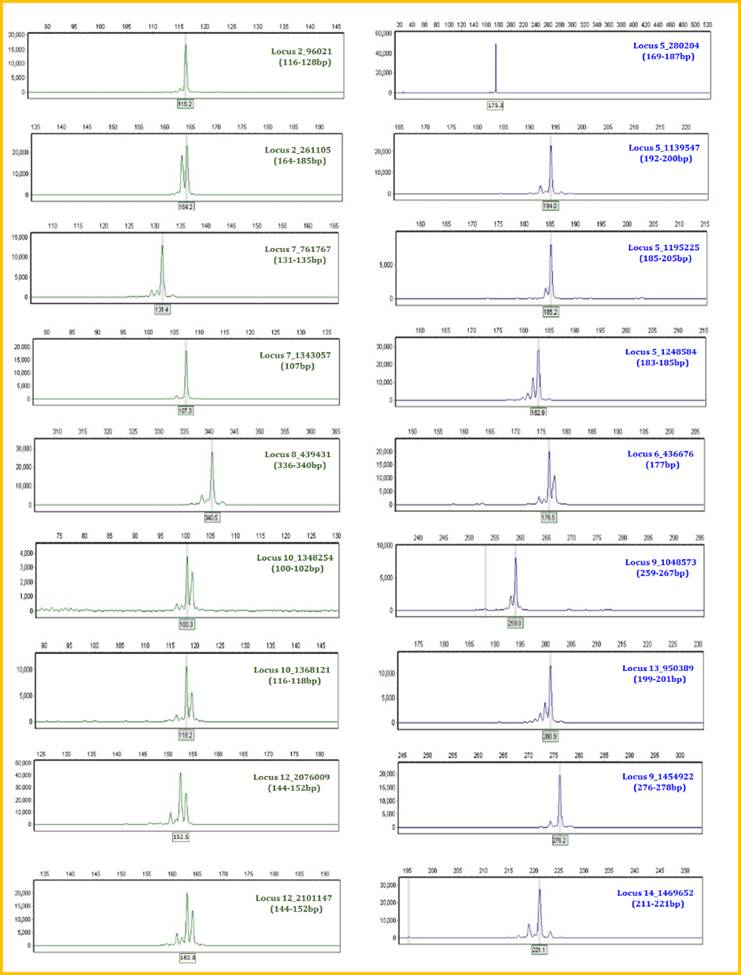

Supplement: S1 Fig — The y-axis corresponds to fluorescence intensity (arbitrary units) and the x-axis is the PCR product length in base pairs (bp). The amplitude of the each peak in base pairs (bp) is shown in boxes underneath the peaks. The range of allele sizes for these small datasets is also given for each locus. (JPG) [file pntd.0004252.s005.jpg]
